# Preserved Function of Circulating Invariant Natural Killer T Cells in Patients With Chronic Hepatitis B Virus Infection: Erratum

**DOI:** 10.1097/01.md.0000469903.81675.69

**Published:** 2015-07-17

In the article “Preserved Function of Circulating Invariant Natural Killer T Cells in Patients With Chronic Hepatitis B Virus Infection”,^1^ which appeared in Volume 94, Issue 24 of *Medicine*, the abbreviation IC was incorrectly defined as immune carrier. It should have been defined as inactive carrier. The article has since been corrected online.
